# Learning to modulate one's own brain activity: the effect of spontaneous mental strategies

**DOI:** 10.3389/fnhum.2013.00695

**Published:** 2013-10-18

**Authors:** Silvia E. Kober, Matthias Witte, Manuel Ninaus, Christa Neuper, Guilherme Wood

**Affiliations:** ^1^Department of Psychology, University of GrazGraz, Austria; ^2^Laboratory of Brain-Computer Interfaces, Institute for Knowledge Discovery, Graz University of TechnologyGraz, Austria

**Keywords:** neurofeedback, mental strategies, sensorimotor rhythm, gamma, EEG, implicit learning

## Abstract

Using neurofeedback (NF), individuals can learn to modulate their own brain activity, in most cases electroencephalographic (EEG) rhythms. Although a large body of literature reports positive effects of NF training on behavior and cognitive functions, there are hardly any reports on how participants can successfully learn to gain control over their own brain activity. About one third of people fail to gain significant control over their brain signals even after repeated training sessions. The reasons for this failure are still largely unknown. In this context, we investigated the effects of spontaneous mental strategies on NF performance. Twenty healthy participants performed either a SMR (sensorimotor rhythm, 12–15 Hz) based or a Gamma (40–43 Hz) based NF training over ten sessions. After the first and the last training session, they were asked to write down which mental strategy they have used for self-regulating their EEG. After the first session, all participants reported the use of various types of mental strategies such as visual strategies, concentration, or relaxation. After the last NF training session, four participants of the SMR group reported to employ no specific strategy. These four participants showed linear improvements in NF performance over the ten training sessions. In contrast, participants still reporting the use of specific mental strategies in the last NF session showed no changes in SMR based NF performance over the ten sessions. This effect could not be observed in the Gamma group. The Gamma group showed no prominent changes in Gamma power over the NF training sessions, regardless of the mental strategies used. These results indicate that successful SMR based NF performance is associated with implicit learning mechanisms. Participants stating vivid reports on strategies to control their SMR probably overload cognitive resources, which might be counterproductive in terms of increasing SMR power.

## Introduction

Using neurofeedback (NF), individuals can learn to modulate their own brain activity. In NF, healthy, age appropriate brainwave activity is rewarded with visual, auditory or even tactile stimulation. In contrast, undesirable patterns of activity are ignored or punished (Coben and Evans, 2010). When participants become successful in regulating their own brain activity, e.g., voluntarily increase specific EEG frequency bands, improvements in cognition and behavior usually follow (Kotchoubey et al., 1999; Wolpaw et al., 2002; Gruzelier and Egner, 2005; Kübler et al., 2005; Kübler and Kotchoubey, 2007; Kropotov, 2009; Coben and Evans, 2010). Hence, there is strong evidence for positive effects of NF training on behavior and cognitive functions. However, researchers have different opinions about underlying mechanisms and processes leading to successful NF performance. There are hardly any reports on how participants can successfully learn to gain control over their own brain activity. In the present study, we addressed this question by focusing on the effects of different mental strategies on NF performance.

Brain signals can be used as a control signal for a brain computer interface (BCI) or to provide NF to participants (LaConte, 2011). Although NF and BCI applications are effective in the rehabilitation and therapy of many disorders, a substantial proportion of participants fail to gain significant control over their brain signals even after repeated training sessions. About 15–30% of potential BCI or NF users cannot attain control over their own EEG (Allison and Neuper, 2010; Blankertz et al., 2010). In the BCI community, the inability to use BCI applications is called “BCI-illiteracy phenomenon” (Blankertz et al., 2010). There are different attempts to explain this phenomenon. In some users of NF or BCI feedback applications, neuronal systems needed for voluntary control might not produce electrical activity detectable on the scalp. Although the necessary neuronal populations are presumably healthy and active in these participants, the activity they produce may not be detectable by a particular neuroimaging method, such as EEG. Another reason might be that some participants produce excessive muscle artifact, which might disturb the feedback signal and hamper the learning effect (Allison and Neuper, 2010). To find possible predictors of the BCI-illiteracy phenomenon, the interest in inter-individual differences in BCI or NF performance is rising. In this context, some researchers found neurophysiological predictors of NF or BCI performance (Neumann and Birbaumer, 2003; Kübler et al., 2004; Blankertz et al., 2010; Halder et al., 2013a,b), others found that psychological factors such as “locus of control” (LOC), degree of concentration, mood, mastery confidence, or motivation can predict NF and BCI performance to some extent (Burde and Blankertz, 2006; Nijboer et al., 2008; Kleih et al., 2010; Hammer et al., 2012; Witte et al., 2013). However, the definite reasons why some people fail to gain significant control over their own brain signals are still unknown.

A first step to identify parameters of success to gain control over one's own brain activity is to define how regulation of physiological parameters such as EEG activity might be learned. In this context, Hammer et al. (2012) defined three different models: The first and in the NF literature most frequently mentioned model is operant conditioning. Operant learning declares that the occurrence of a positively reinforced behavior will increase (Skinner, 1945). Consequently, in NF studies correct or desired brain responses are positively reinforced by getting reward points, a smiling face, etc. (Kübler et al., 1999; Leins et al., 2007; Weber et al., 2011). In NF studies, participants can freely choose different mental strategies to control their own brain activity, which results in trial-and-error learning. By means of trial-and-error, participants use diverse strategies and repeat them when positively reinforced (Curran and Stokes, 2003; Hammer et al., 2012). The second model suggests that the feedback-learning of physiological parameters is comparable with motor learning (Lang and Twentyman, 1976). In a biofeedback study by Lang and Twentyman (1976), participants should learn to control their own heart rate. The authors proposed that the ability to control one's own heart rate could be conceptualized as the acquisition of motor learning. According to Lang and Twentyman, the voluntary control over cardiovascular processes requires a well-organized sequence of activities, movements and symbolic information. These should be the same processes necessary to hit for instance a tennis ball correctly. This model might be transferred to self-regulation of other physiological parameters such as EEG parameters as well (Hammer et al., 2012). Kropotov (2009) also compared the learning procedure during NF training with the technique how we learn motor skills such as to drive a bicycle (Kropotov, 2009). The third model of how to regulate one's own brain activity is the dual process theory (Lacroix and Gowen, 1981; Lacroix, 1986). This theory describes learning as an interaction of feed-forward and feed-back processes. The naïve learner searches for an effective strategy. This cognitive process needs a high degree of attentional resources due to trial-and-error learning. The decision for a mental strategy depends on the provided instruction. If the learner already has an effective strategy, it will be maintained and improved. However, if the learner has no effective strategy, the novice has to design a new motor activation-model. If this model turns out to be successful it will be maintained and improved. In a final step, this process becomes automatic. The learned skill is stored in the implicit memory and its retrieval requires no consciousness any more (Strehl, 2013). According to Lacroix and colleagues, the instruction plays a central role in the learning success. In line with this assumption, Neuper et al. (2005) found differences in the EEG patterns during motor imagery depending on the instruction provided to the participants (Neuper et al., 2005). Participants were told to either imagine a hand-movement kinaesthetically (feeling of movement) or visually (seeing the movement in their mind's eye). Only for the kinaesthetic imagery, EEG activity over sensorimotor areas was comparable to that of actual movement (Neuper et al., 2005). In contrast to typical BCI applications, where very specific instructions can be transmitted to participants straightforwardly (Curran and Stokes, 2003; Friedrich et al., 2012, 2013), the exact instruction given by the experimenters to the participants in NF studies are hardly described in detail (Hoedlmoser et al., 2008).

In summary, one of the core features of successful NF performance is the used mental strategy. However, the effects of spontaneous mental strategies on NF performance are scarcely investigated. A study by Nan et al. (2012) is one of the rare examples investigating different mental strategies used to gain control over the own EEG activity in a NF application. In that study, participants were instructed to employ any strategies they like in an individual Alpha NF training, but they should use only one strategy in each trial. After each trial, participants wrote down the strategy they used to control their own EEG and rated how successful this strategy for self-regulating their EEG was. Nan et al. (2012) reported the subjective self-rating scores of efficiency for each strategy. This analysis of self-comments showed that what is an useful strategy varies among individuals and that the most successful strategies when training their individual Alpha rhythm were related to positive thinking such as thoughts about lover, friend and family (Nan et al., 2012). Moreover, Angelakis et al. (2007) reported similar findings when participants learned to increase their individual Peak Alpha Frequency (PAF) or their Alpha power. Particularly, Alpha amplitude was higher when participants reported to have positive thoughts during training and when they reported that they thought of nothing particular, or had a blank mind during NF training (Angelakis et al., 2007).

In the present NF study, participants were instructed to employ any mental strategy they wanted to increase either their own sensorimotor rhythm (SMR, 12–15 Hz) or high-frequency EEG rhythms (Gamma, 40–43 Hz). The SMR generally emerges when one is motionless yet remains attentive (Sterman, 1996, 2000; Serruya and Kahana, 2008). Hence, one could assume that the best mental strategy to increase SMR power is to be mentally focused and physically relaxed. Several NF studies provide evidence that healthy individuals are able to learn how to *increase* their own SMR amplitude (Tansey and Bruner, 1983; Tansey, 1984; Tinius and Tinius, 2000; Vernon et al., 2003; Egner et al., 2004; Schabus et al., 2004; Hoedlmoser et al., 2008; Doppelmayr and Weber, 2011). However, none of these studies analyzed formally the mental strategies employed by the participants to control SMR power. In BCI studies, amplitude *reductions* of the SMR rhythm can be voluntarily controlled by most participants, for instance by using motor imagery strategies such as imaging a hand or foot movement (Kübler et al., 2005; Blankertz et al., 2010). Though, motor imagery leads to decreased SMR amplitude over the motor cortex (Pfurtscheller and Neuper, 1997; Pfurtscheller and Lopes da Silva, 1999). Voluntary increase in SMR power cannot be reached by motor imagery strategies, which is required in most SMR based NF applications (Tansey and Bruner, 1983; Tansey, 1984; Tinius and Tinius, 2000; Vernon et al., 2003; Egner et al., 2004; Schabus et al., 2004; Hoedlmoser et al., 2008).

A second group of participants should learn to voluntarily increase their Gamma (40–43 Hz) power. Studies on meditators showed that Gamma power was intensified during meditation, and that Gamma is apparently associated with feelings of kindness and compassion (Banquet, 1973; Lutz et al., 2004; Rubik, 2011). Some NF studies could show that people are able to alter the power in the Gamma frequency band voluntarily by means of real-time feedback (Bird et al., 1978; Keizer et al., 2010a,b; Rubik, 2011). However, these NF studies do not provide any concrete explanations or descriptions on how people actually managed to increase or decrease Gamma power voluntarily (Keizer et al., 2010a,b). A study by Rubik (2011) is one of the rare examples aiming to explore inner experiences associated with increased production of Gamma brainwaves in an initial NF experience (Rubik, 2011). Increased Gamma power during an initial NF training session was associated with positive emotions of happiness and love, along with reduced stress. On the basis of the NF study by Rubik (2011) and the studies on meditators one could conclude that the best mental strategy to modulate EEG Gamma activity voluntarily might be to produce positive feelings such as happiness, love, kindness, or compassion.

The aim of the present study was to investigate the effects of spontaneous mental strategies on gaining control over SMR or Gamma activity during repeated NF training, respectively. To this end, naïve NF users wrote down their mental strategies after the first and last NF training session. According to the literature, we expect that different mental strategies have different effects on NF performance. For instance, positive thoughts and thinking on nothing particular should lead to an increased NF performance compared to negative thoughts (Angelakis et al., 2007; Rubik, 2011; Nan et al., 2012). Furthermore, we wanted to examine whether the success of different mental strategies is frequency specific, or if diverse mental strategies lead to the same NF training outcome in the SMR and Gamma group. Since there are no prior NF studies linking concrete mental strategies to voluntary control over SMR or Gamma power, it remains unclear whether similar results will be obtained for the SMR and Gamma NF training or not.

## Materials and methods

### Participants

A total of 20 healthy participants (10 males and 10 females, aged 40–63 years: Mean age = 46.40 years, *SE* = 1.71) took part in this study. All participants were novices for NF- and BCI-experiments. All volunteers gave written informed consent and were paid for their participation (7€ per hour). The ethics committee of the University of Graz, Austria approved all aspects of the present study in accordance to the Declaration of Helsinki. Participants were randomly assigned to one of two NF groups: a SMR group (5 males, 5 females, Mean age = 46.80 years, *SE* = 1.99) and a Gamma group (5 males, 5 females, Mean age = 46.00 years, *SE* = 1.26). The SMR group performed a SMR (12–15 Hz) based NF training. Hence, this group was rewarded whenever their SMR power exceeded a predefined threshold. The Gamma group performed a Gamma (40–43 Hz) based NF training. Therefore, this group was rewarded whenever their Gamma power exceeded a predefined threshold. Participants were not informed about the grouping design, nor did they know that there were different conditions.

### Neurofeedback training

The EEG signal was recorded from Cz channel (according to the international 10–20 EEG placement system), the ground was located at the right mastoid, the reference was placed at the left mastoid. Furthermore, one EOG channel was recorded. Therefore, the positive electrode was placed above and the negative electrode was placed below the left eye. The signals were amplified by a 10-channel system (NeXus-10 MKII, Mind Media BV). The EEG and EOG signals were digitized at 256 Hz and low-pass filtered with 64 Hz.

The NF paradigm was generated by using the software BioTrace+ (Mind Media BV). Ten NF training sessions were carried out within 3 weeks. Each session consisted of seven runs á 3 min each. The first run was a baseline run. In this baseline run participants saw three moving feedback bars on the screen depicting their own EEG activity but were instructed to relax themselves and not to try to control the bars voluntarily. The subsequent six runs were feedback runs, where participants were instructed to voluntarily control the moving bars.

The feedback display contained three moving bars: One big bar in the middle and two smaller bars on the left and right side of the feedback screen. During each three-minute run the feedback bars were continuously moving in a vertical direction. The height of the bar in the middle of the screen reflected absolute SMR (12–15 Hz) band power in real time for the SMR group and absolute Gamma (40–43 Hz) band power in real time for the Gamma group, respectively. The width of the Gamma and SMR band was made identical to prevent possible effects of a band-width difference in the Gamma and SMR band (Keizer et al., 2010a,b). Whenever the band power reached an individual predefined threshold in the feedback runs, the color of this bar changed from red to green and participants were rewarded by getting points, which were also displayed at the feedback screen (reward counter). Furthermore, as a reward auditory feedback was provided by means of a midi tone feedback. When the bar was below the threshold it turned red again, the reward counter stopped and no tone was presented. Participants were instructed to try to voluntarily increase this bar. The threshold for the SMR/Gamma bar was adapted after each run. The mean of the SMR/Gamma power of the previous run was taken as SMR/Gamma threshold in the actual feedback run.

In order to prevent augmentation of the SMR or Gamma signal by muscle artifacts, such as movements or eye blinks, two inhibit-bands were used, represented on the screen by the two smaller vertical moving bars on the left and right side of the display. The small bar on the left side of the feedback screen depicted EEG band power between 4 and 7 Hz indicating eye blinks, and the small bar on the right side depicted EEG band power between 21 and 35 Hz indicating movements and other high frequency disturbances (Doppelmayr and Weber, 2011; Weber et al., 2011). Artifact rejection thresholds were set for each participant individually (mean of baseline run + 1 *SD*), suspending feedback when eye-movements or other muscle activity caused gross EEG fluctuations. Hence, participants were instructed to keep these two bars as small as possible, but they were not told that they could influence the height of these bars by muscle activity or eye-movements. Participants were not rewarded when these two controlling bars were above their related thresholds even when SMR/Gamma was above the individually defined threshold.

### Mental strategy

After the first and the last NF training session, participants were asked which mental strategy they have used to gain control over the moving bars. Before the NF training, we did not prescribe any specific strategies which might be useful to control the bars. Participants were only instructed to be mentally focused and physically relaxed during the NF training in order to avoid producing too many artifacts. Hence, during the NF trainings, participants could utilize any mental strategy they wanted. To help participants finding out the efficient strategy for self-regulating their EEG, they were asked to write down the strategy used and its effect after the first and the last training session.

**Figure 1:**
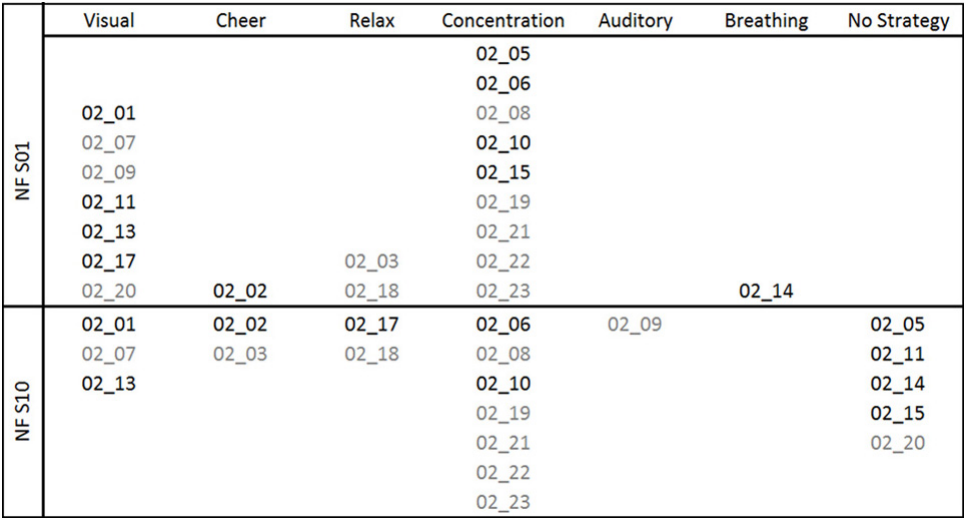
**Mental strategies used during the first (NF S01) and the last (NF S10) NF training session, presented separately for each participant of the SMR (subject code in black font color) and Gamma group (subject code in gray font color)**.

The reported mental strategies were divided into different categories: Visual strategies, auditory strategies, cheering strategies, relaxation, concentration, breathing, and no strategy. The reported mental strategies and the subsequent categorization process are described in TableA1 of the Appendix in more detail. Mental strategies, which were classified as visual strategies, contained imagination of colors or objects. Auditory strategies reflected the imagery of tones or sounds. Participants using cheering strategies tried to increase the SMR/Gamma bar by cheering it on. Others tried to relax as much as possible to increase SMR/Gamma. Concentration strategies refer to focused attention and concentration on the moving bars. Breathing methods were used as well, where participants tried to consciously regulate their breath to gain control over their own EEG. And the last category included all reports in which the participants did not name any specific strategy. In Figure 1, the frequencies of the mental strategies used during the first and the last NF training session are shown, separately for the SMR (black font color) and Gamma group (gray font color). For instance, three participants of the Gamma group used a visual strategy during the first NF session. One of these three participants still used the visual strategy during the last NF session, one of them switched to an auditory strategy and one reported no specific mental strategy during the last training session any more.

After the first NF training session, all NF-naïve participants reported to use a specific mental strategy. After the tenth NF training session, four participants of the SMR group and one participant of the Gamma group reported to have no particular strategy any more. Based on the subjective reports after the last NF session, participants were split up in two groups for subsequent statistical analyses: Participants using mental strategies to control their own EEG activity (SMR strategy group: 2 males, 4 females; Gamma strategy group: 4 males, 5 females) and participants describing no specific mental strategy to control their own EEG activity after gaining some NF experience (SMR no strategy group: 3 males, 1 female; Gamma no strategy group: 1 male).

### EEG data analysis

Data preprocessing and analysis were performed with the Brain Vision Analyzer software (version 2.01, Brain Products GmbH, Munich, Germany). Ocular artifacts such as eye blinks were manually rejected by visual inspection based on the information about EOG activity provided by the EOG channel. After ocular artifact correction, automated rejection of other EEG artifacts (e.g., muscles) was performed (Criteria for rejection: >50.00 μV voltage step per sampling point, absolute voltage value >±120.00 μV). All data points with artifacts were excluded from the EEG analysis (15% of data).

For the EEG data analysis, absolute SMR (12–15 Hz) and Gamma (40–43 Hz) band power was extracted by means of complex demodulation (Brain Products GmbH, 2009). The extracted power values were averaged over the whole artifact free training runs in one session. For statistical analyses and better comparability of the data, SMR and Gamma power values were *z*-transformed.

## Results

### Neurofeedback performance: mental strategy vs. reporting no specific strategy

#### SMR group

**Figure 2:**
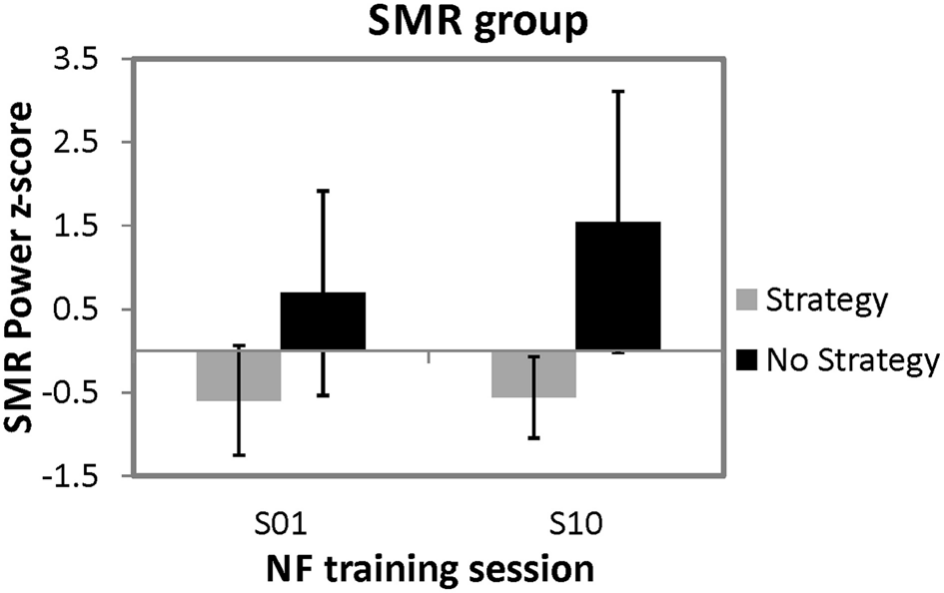
**Means and standard deviations of *z*-transformed SMR power (12–15 Hz) values during the first and tenth NF training session, presented separately for participants reporting to use a specific mental strategy in the tenth NF session (strategy group) and participants reporting no specific mental strategy in the tenth NF session (no strategy group)**.

In order to investigate the effects of spontaneous mental strategies on SMR based NF performance, a 2 × 2 univariate repeated measures analysis of variance (ANOVA) with the between subject factor strategy group (strategy group vs. no strategy group) and the within-subject factor time (first vs. last NF training session) was applied for the dependent variable *z*-transformed SMR power values. The ANOVA revealed a significant main effect of time [*F*_(1, 8)_ = 8.81, *p* < 0.05, η^2^ = 0.52] and a significant main effect of strategy group [*F*_(1, 8)_ = 7.69, *p* < 0.05, η^2^ = 0.49]. Overall, SMR was higher in the last (*M* = 0.49 *z*-score, *SD* = 1.05) compared to the first NF training session (*M* = 0.05 *z*-score, *SD* = 0.93), and the no strategy group (*M* = 1.12 *z*-score, *SD* = 1.50) showed higher SMR values than the strategy group (*M* = −0.58 z-score, *SD* = 1.22). Moreover, the interaction effect strategy group^*^time [*F*_(1, 8)_ = 7.41, *p* < 0.05, η^2^ = 0.48] was significant, too. Posttests showed that the two strategy groups did not differ in their SMR power during the first NF training session [*t*_(8)_ = −2.18, *ns*.]. In contrast, during the last NF training session, participants reporting no specific strategy showed significant higher SMR power values than participants still using a specific mental strategy [*t*_(8)_ = −3.15, *p* < 0.05]. Furthermore, participants still using a specific mental strategy in the last NF training session showed no significant changes in SMR power between the first and the last training session [*t*_(5)_ = −0.38, *ns*.], whereas the no strategy group showed a trend toward an increased SMR power during the last compared to the first training session [*t*_(3)_ = −2.46, *p* = 0.09]. In Figure 2, means and standard deviations of *z*-transformed SMR power values during the first and last NF session are illustrated, separately for both groups.

**Figure 3:**
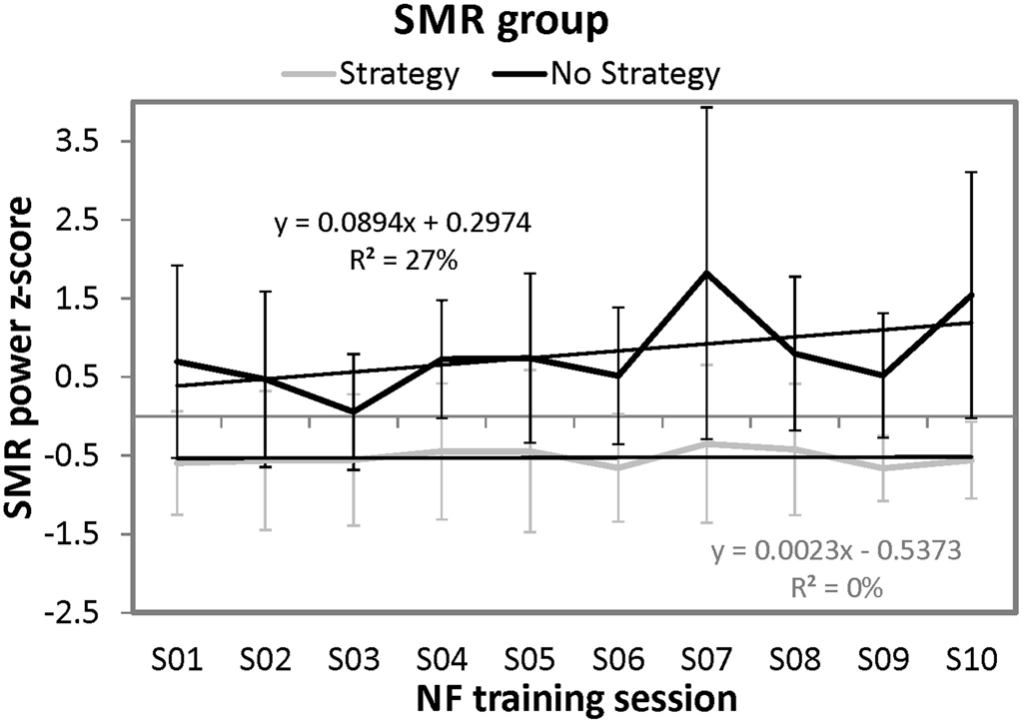
**Means and standard deviations of *z*-transformed SMR (12–15 Hz) power (NF performance) over the ten NF training sessions, presented separately for participants reporting to use a specific mental strategy in the tenth NF session (strategy group) and participants reporting no specific mental strategy in the tenth NF session (no strategy group) of the SMR group and the results of the regression analyses**.

In order to analyze the time course of SMR power over the ten training sessions in more detail, we conducted regression analyses separately for the strategy and the no strategy group (predictor variable = session number; dependent variable = *z*-transformed SMR power). For the no strategy group, the regression model was by trend significant [*F*_(1, 8)_ = 3.34, *p* = 0.10]. With this regression model, 27.09% of variance of SMR power over the training sessions can be explained. When analyzing the time course of SMR power over the ten sessions separately for each participant of the no strategy group, all of them (i.e., 100%) showed a positive regression slope of the learning curve. In contrast, the regression model for the strategy group was not significant. Furthermore, we compared the regression slopes of the learning curves over the ten NF sessions between the strategy group and the no strategy group. The no strategy group showed significant higher positive slopes (*M* = 0.089, *SD* = 0.035) than the strategy group (*M* = 0.002, *SD* = 0.040) [*t*_(8)_ = −3.52, *p* < 0.01]. In Figure 3, the NF performance over all ten NF training sessions (means and standard deviations) is depicted for both groups. The no strategy group shows a linear increase in SMR power over the ten training sessions, whereas the strategy group shows no prominent changes in SMR power over all training sessions.

Note that participants of the SMR group did not show any linear increase or decrease in Gamma power over the 10 NF training sessions.

#### Gamma group

To evaluate the effects of mental strategies on Gamma based NF performance, the same ANOVA as for the SMR group was applied for the dependent variable *z*-transformed Gamma power values. This ANOVA revealed no significant results. The results of the ANOVA should be interpreted with caution because only one participant formed the no strategy group. Therefore, we applied special *t*-tests comparing an individual's test score (single participant of the no strategy group) against norms derived from small samples (strategy group) (Crawford and Howell, 1998; Crawford and Garthwaite, 2002; Crawford et al., 2010). In the first [*t*_(8)_ = −0.27, *ns*.] and the last NF training session [*t*_(8)_ = −0.52, *ns*.] this single participant of the no strategy group did not differ significantly in his *z*-transformed Gamma values from the strategy group.

**Figure 4:**
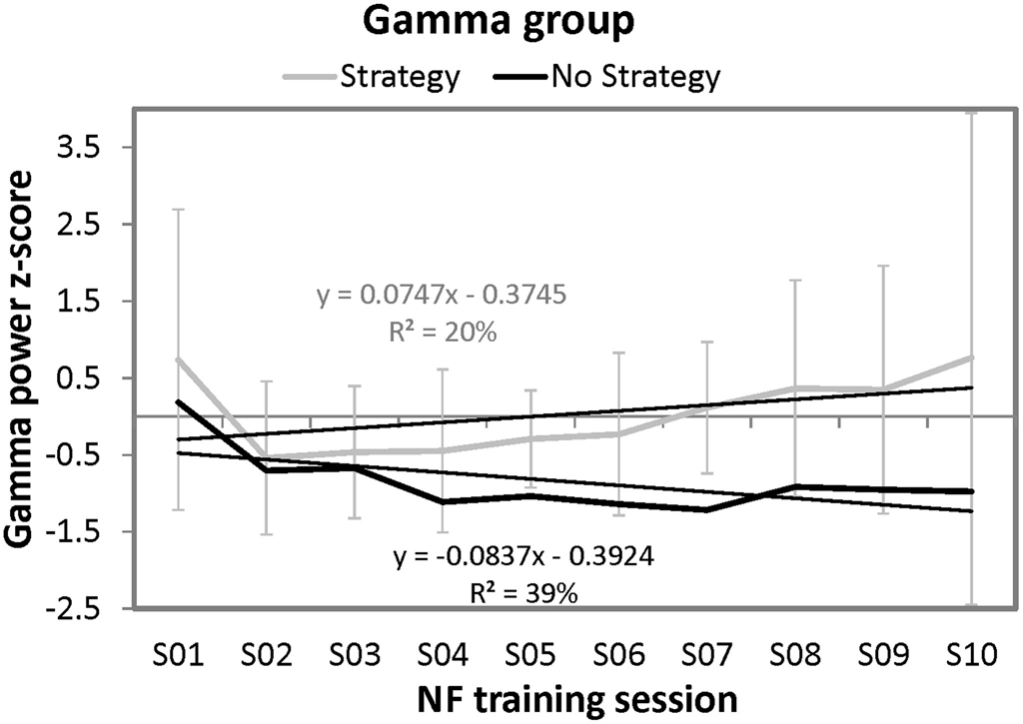
**Means and standard deviations of *z*-transformed Gamma (40–43 Hz) power (NF performance) over the ten NF training sessions, presented separately for participants reporting to use a specific mental strategy in the tenth NF session (strategy group) and participants reporting no specific mental strategy in the tenth NF session (no strategy group) of the Gamma group and the results of the regression analyses**. Note that only standard deviations for the strategy group are plotted since only one participant was present in the no strategy group.

Furthermore, the same regression analyses were conducted as for the SMR group to examine the time course of Gamma power over the ten NF trainings. For the strategy group, this regression analyses did not reveal significant results. However, the regression model for the single participant of the no strategy group was significant by trend [*F*_(1, 8)_ = 5.14, *p* = 0.05]. 39.09% of variance in Gamma power could be explained by session number. In contrast to the no strategy group of the SMR NF training group, the single participant of the Gamma group that reported no specific mental strategy to increase Gamma power voluntarily showed a linear decrease in NF training performance over the ten sessions. When comparing the slope of the single participant reporting no strategy with the strategy group's slopes, no significant differences could be found [*t*_(8)_ = −1.21, *ns*]. (Crawford and Garthwaite, 2004). In Figure 4, the NF performance over all ten NF training sessions is depicted for both groups.

Participants of the Gamma group did not show any linear increase or decrease in SMR power over the 10 NF training sessions.

### Neurofeedback performance: diverse mental strategies

**Figure 5:**
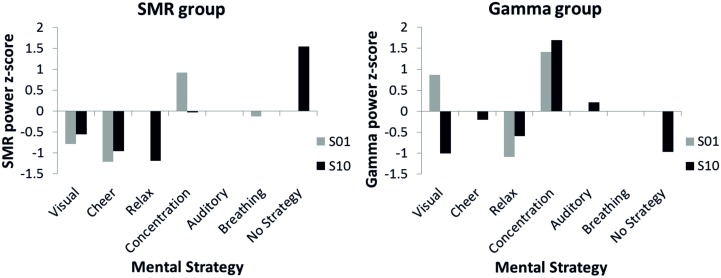
***Z*-transformed SMR power for the SMR group (left panel) and *z*-transformed Gamma power for the Gamma group (right panel) during first (gray bars) and tenth (black bars) NF training session, presented separately for different mental strategies used to control one's own EEG signal**. Note that no error bars are plotted since only single participants were present in several categories.

The effects of the distinct mental strategies on the NF performance (SMR or Gamma power) are shown in Figure 5. In the first NF training session, where all participants reported using a mental strategy, for both the SMR and the Gamma group the most effective strategy seemed to be concentration. In the last NF training session, four participants of the SMR group reported to use no specific strategy any more to gain control over the EEG and six participants still described specific mental strategy in detail in the introspective report. The SMR based NF performance was highest in the no specific strategy condition during the last SMR based NF training session. In contrast, for the Gamma group reporting no specific mental strategy was not the most successful strategy to increase Gamma power voluntarily. The Gamma group was most successful when using the concentration strategy in both the first and the last NF session. Hence, the concentration strategy did not lead to a linear increase in Gamma power over the training sessions but rather to a constantly high Gamma power. The concentration strategy seems to be useful to increase SMR, too. SMR power was second highest for the concentration strategy in the tenth training session and highest during the first session. The relaxation strategy turned out to be the least effective mental strategy to increase SMR or Gamma power voluntarily. The breathing strategy was the second most effective in the first SMR based NF training session. However, nobody used this strategy at the end of the training.

## Discussion

The present work focused on the effects of spontaneous mental strategies used to control the EEG activity during NF training. NF users reported their spontaneous strategies to increase either their SMR (12–15 Hz) or Gamma (40–43 Hz) amplitude after the first and tenth NF training session. The usage of different mental strategies only affect SMR based NF performance but not Gamma related NF performance. After the first NF training session, all participants reported to use different types of mental strategies. After the last NF training session, some NF users reported no longer a particular strategy to control their SMR or Gamma power. In the SMR group, these participants showed a steadily increasing NF performance over ten NF training sessions. In contrast, participants still using a specific mental strategy in the last SMR based NF training session showed no significant improvements over the NF training sessions. Hence, our results show that different mental strategies have different effects on SMR based NF performance, but not on Gamma based NF performance. In the following paragraphs, these results are discussed in more detail.

In the first NF training session, all NF-naïve participants spontaneously verbalized a mental strategy to obtain control over their EEG power. This piece of evidence is in line with the assumption that learning to control one's own brain activity is associated with trial-and-error learning. By means of trial-and-error, the participants use diverse strategies and repeat them when positively reinforced (Curran and Stokes, 2003; Hammer et al., 2012). Though, after gaining some NF experience, four out of ten NF users of the SMR group and one participant of the Gamma group did not explicitly name any kind of specific mental strategy to control the feedback bar depicting their own EEG activity. This result is in line with the dual process theory (Lacroix and Gowen, 1981; Lacroix, 1986), which describes learning as an interaction of feed-forward and feed-back processes. In a first step, the NF user searches for an effective strategy. Therefore, all participants reported to use diverse mental strategies during the first NF session. After the NF user has found an effective strategy, this strategy will be maintained and improved and the strategy will become automatic. The learned skill to control the own SMR activity is stored in the implicit memory and its retrieval requires no consciousness any more (Strehl, 2013). Hence, those participants who did not report any specific mental strategy after the last NF session might have developed such an automatic mechanism. Nevertheless, it is also possible that the strategies verbalized by participants are not causally but only circumstantially connected with NF learning. Rather, the mechanisms to increase SMR becoming increasingly automatic during NF training may not correspond functionally to the content of the strategies verbalized. If this is correct, the role of explicit learning mechanisms in NF may be more limited than that predicted by a dual process account.

To investigate the effects of spontaneous mental strategies on NF performance, participants of the SMR and Gamma group were divided into two sub-groups, respectively: one group of participants still using a specific mental strategy after ten NF training sessions, which was termed “strategy group,” and participants who did not verbalize mental strategies after ten NF training sessions formed the second group, the so called “no strategy group.” In the first NF training session, these two strategy groups did not differ significantly in their power values during the feedback training neither in the SMR nor in the Gamma group. This result seems to be obvious, because after the first session, all participants quoted that they have used a specific mental strategy during the NF training session. Hence, all participants spent cognitive resources and mental effort to gain control over their EEG signal. Consequently, SMR/Gamma power did not differ between the two strategy groups and the NF performance was comparable across subgroups during the first NF training session.

However, in the SMR group, the NF performance differed between the two strategy groups over the NF training course. The no strategy group showed a steady linear increase in SMR power over the ten NF training sessions as indicated by the regression analyses, which cannot be seen in the strategy group (Figure 3). During the last NF training session, participants reporting no particular strategy showed significantly higher SMR power values than participants reporting a specific mental strategy. Furthermore, participants still using a specific mental strategy in the last NF training session showed no significant changes in SMR power between the first and the last NF training session, whereas the no strategy group showed higher SMR power values at the end of the training compared to the first training session. These results further indicate that the no strategy group developed an automatic mechanism to control their SMR over the NF training sessions. Participants trying to control their SMR by using a specific mental strategy probably overload cognitive resources, which might be counterproductive in terms of increasing SMR power, since the sensorimotor rhythm in the EEG is associated with a state of physical relaxation and simultaneous mental focusing (Sterman, 1996, 2000; Nijboer et al., 2008; Serruya and Kahana, 2008). Prior NF studies investigating the effects of mental strategies on NF performance support our findings (Angelakis et al., 2007; Nan et al., 2012). Especially, Angelakis et al. (2007) reported on the positive effects of “thinking on nothing particular” on NF performance, which might be compatible with the “no strategy” technique used by some of our participants in the last NF training session. Although the participants of the no strategy group did not verbalize any specific mental strategy to gain control over their EEG, we do not know exactly what they were doing during the last NF training session to increase SMR. Did they have a totally “blank mind,” or did they think on nothing particular, did they think on friends or something that had happened the day before? However, we do know that they did not spend too much effort in using different mental strategies, forcing to gain control over the own EEG. Probably, participants of the no strategy group automatized the skill of modulating the own EEG activity and therefore they did not need any specific mental strategies any more after repeated NF training sessions. This is in line with the assumption that the learned skill to successfully control the own SMR activity is stored in the implicit memory (Strehl, 2013). In contrast, participants that used a mental strategy described these strategies relatively detailed in the introspective report. This might also be a sign that participants of the strategy group spent too much mental effort and overloaded cognitive resources, leading to no improvements in SMR based NF performance.

Although not significant, the no strategy group presented a numerical advantage of about 1 *z*-score in SMR power in comparison to their peers from the strategy group in the first session of training (see Figure 3). This result might indicate that participants of the no strategy group have a predisposition to better up-regulate SMR activity. This predisposition manifests itself in two different ways: Firstly, participants of the no strategy group show higher levels of SMR power independently of training. Secondly, these same participants are more able to up-regulate their SMR power levels over the course of training. Moreover, these participants are also prone to report less explicit strategies after training, which might be indicative of stronger reliance upon implicit learning mechanisms largely independent of overt mental strategies. Further studies are needed to investigate the relation between the spontaneous use of mental strategies and SMR training success in more detail.

Importantly, not all mental strategies seem to be equally ineffective. In the first NF training session, where all participants verbalized a mental strategy, the most effective strategy seemed to be concentration. Hammer et al. (2012) also mentioned that the degree of concentration plays an important role in feedback studies. These authors found that the ability to concentrate on the feedback task is supportive for BCI performance because distracting stimuli can be better ignored. Furthermore, the authors speculate that performing a feedback task requires self-regulatory capacities to focus on and comply with the task despite possibly distracting thought (Hammer et al., 2012). Astonishingly, relaxation strategies turned out to be the least effective mental strategies to increase SMR power beside cheering and visual strategies. It is possible that the state of relaxation was reached anyway but in a less explicit and less controlled way. That the strategy to “relax” disappears from the focus of attention and from the focus of cognitive control employed may have helped that relaxation really happened. The breathing strategy was the second most effective in the first NF training session. However, nobody explicitly reported this strategy at the end of the training. Probably, the strategy of breathing consciously led to physical relaxation too, which might have increased SMR power. Summing up, looking at the effects of the different mental strategies on NF performance reveals that they have diverse effects. However, the most effective strategy to increase SMR voluntarily was not to be able no name anyone.

In sharp contrast, reports on spontaneous mental strategies had no specific effects on Gamma based NF training performance. In the Gamma group, only one participant reported to use no particular strategy any more to control the feedback bar during the last NF training session, which was counterproductive in terms of increasing Gamma power (see Figure 4). Hence, our findings do not support the findings by Keizer et al. (2010a,b) and Bird et al. (1978) who could show that people are able to modulate the power in the Gamma frequency band voluntarily (Bird et al., 1978; Keizer et al., 2010a,b; Rubik, 2011). Gamma power seems to be associated with meditative states, such as positive feelings of happiness, love, kindness, or compassion (Banquet, 1973; Lutz et al., 2004; Rubik, 2011). None of our participants reported to use such meditative mental strategies to modulate Gamma power voluntarily, which might be the reason why the Gamma group showed no changes in NF performance.

One critical issue in NF studies is the instruction provided by the experimenter (Lacroix and Gowen, 1981; Lacroix, 1986; Neuper et al., 2005; Hammer et al., 2012). In the present study, we did not prescribe any specific mental strategies which might be useful to control the feedback bars, as in the majority of NF studies. However, we gave our participants a minimal instruction, telling them to try to be mentally focused and physically relaxed during the NF training in order to increase their EEG amplitude (Leins et al., 2007; Serruya and Kahana, 2008). During the NF training sessions, participants regularly asked how to control their EEG voluntarily and if there are any useful strategies. But we did not give them any further instructions. Instructions given to the participants in prior NF studies are scarcely described. For instance, NF users were encouraged to look for themselves for appropriate strategies like physiological relaxation combined with positive mental activity (Raymond et al., 2005; Hoedlmoser et al., 2008; Gevensleben et al., 2009; Gruzelier et al., 2010; de Zambotti et al., 2012). Others explained the feedback loop and the rationale of the procedure in detail to their participants prior to taking part in the NF study (Vernon et al., 2003; Kropotov et al., 2005; Dempster and Vernon, 2009). In his NF review, Kropotov (2009) also addressed the question how to guide NF users to achieve the task in the most efficient way. He summarized that some practitioners prefer not to give any instructions to their participants by simply saying “Just do it.” Others give instructions depending on the type of NF procedure: relaxation or activation (Kropotov, 2009).

In conclusion, in prior NF studies no standard instructions have been used. Our analyses of the spontaneous mental strategies used to control one's own brain activity revealed that participants are trying out diverse mental strategies at the beginning of the training. However, after gaining some NF experience, some participants do not verbalize specific mental strategies any more probably because of the development of automatic regulation skills. These participants are most successful in increasing SMR power voluntarily. Hence, we conclude that explicit instructions on how to control the feedback bar might be counterproductive in terms of impartiality and effortlessness during the training. Of course participants should be informed about the study process to some extent, but explaining the detailed feedback loop might stress participants too much since then they know how it should work theoretically. When these informed NF users are not successful from the beginning, they might become frustrated and probably start spending too much mental effort by using diverse mental strategies, and this may hamper performance and further learning. Hence, in accordance with our findings, we would suggest that the best instruction for future SMR based NF training studies is to tell the participants not trying too hard and to “just do it.”

One important limitation of the current study is the sample size because it constrains the generalization of the present findings to other contexts. It is possible that other spontaneously verbalized strategies not occurring in the present study are more effective. Moreover, the contents of individual verbalizations were summarized using criteria defined *post-hoc* by the experimenter. To which extent the list of strategies spontaneously verbalized in NF studies has to be complemented is a question for future studies.

## Conclusion

Here we show that mental strategies used to gain control over the own brain activity play an important role in successful NF performance, especially for SMR based NF performance. Distinct mental strategies have different effects on SMR based NF performance. However, not being able to name a specific one seems to be most effective, indicating the development of more automatic regulation mechanisms. More automatic processes seem to lead to a focused but relaxed mental state, which is beneficial when trying to increase SMR power voluntarily. These results have practical implications on future NF studies and provide guidelines for the instruction of NF users.

### Conflict of interest statement

The authors declare that the research was conducted in the absence of any commercial or financial relationships that could be construed as a potential conflict of interest.

## Appendix

After the first and the last NF training session, participants wrote down the mental strategies they have used to control the feedback bars. Some of these introspective reports were very detailed descriptions of the used mental strategies, whereas others comprised only a few catchwords. In TableA1, these introspective reports are listed and the subsequent classification of these subjective descriptions into the different categories (visual strategies, auditory strategies, cheering strategies, relaxation, concentration, breathing, and no strategy) is specified, too. In most cases, the classification of the subjectively described strategies was unambiguous. However, when participants reported more than one strategy (e.g., participant 02_13, who reported visual strategies and relaxation in between) the most salient strategy was taken as background for the classification (e.g., for participant 02_13, the reported subjective strategy was categorized as visual strategy, since the visual strategy was more precisely described than the relaxation strategy).

**Table A1:** **Introspective reports of the used mental strategies to gain control over the feedback bars during the first (NF S01) and last (NF S10) neurofeedback training session, presented separately for each participant and the subsequent classification into the different strategy categories (visual strategies, auditory strategies, cheering strategies, relaxation, concentration, breathing, and no strategy)**.

**Code**	**NF group**	**Mental strategy NF S01**		**Mental strategy NF S10**	
		**Introspective report**	**Categorization**	**Introspective report**	**Categorization**
02_01	SMR	Visual imagination of a coffee cup standing on the bars on the left and the right side of the screen. The feedback bar in the middle of the screen was deemed as a roller blind.	Visual	Visual imagination of a green lawn, counting the reward points.	Visual
02_02	SMR	Issue commands on the bar in the middle of the screen.	Cheer	Cheering on the bars.	Cheer
02_03	Gamma	Fixation of the bar in the middle and trying to “switch off” any thoughts while total relaxation. Ignored bars on the left and the right side of the screen.	Relax	Cheering on the bars like cheering on the own kids.	Cheer
02_05	SMR	Changing between different levels of concentration, from very high to very low.	Concentration	No strategy used because any mental effort did not lead to successful results.	No strategy
02_06	SMR	Concentration on the bar in the middle of the screen.	Concentration	Concentration on the bar in the middle of the screen.	Concentration
02_07	Gamma	Visual focusing, visual lifting of the bar in the middle of the screen.	Visual	Visual focusing of a point on the screen.	Visual
02_08	Gamma	Concentration on the bar in the middle of the screen and ignoring the left and right bar.	Concentration	Concentration on the bar in the middle of the screen and ignoring the left and right bar.	Concentration
02_09	Gamma	Visual imagination of a specific scene of a movie (imagination of an actor and a ship being moved over a mountain).	Visual	Imagination of different music genres (except folk music).	Auditory
02_10	SMR	Fixation of bar in the middle of the screen and concentration on visual and auditory feedback.	Concentration	Concentration on the upper part of the bar in the middle of the screen.	Concentration
02_11	SMR	Visual focusing of the red and green bars on the screen to allow only green and no red bars.	Visual	No strategy used.	No strategy
02_13	SMR	Visually following the moving bar in the middle of the screen.	Visual	Visually focusing the moving bar in the middle of the screen and whenever this bar turned red for too long this bar was ignored. Relaxation in between.	Visual
02_14	SMR	Breathing to provide the brain with oxygen.	Breathing	No strategy used.	No strategy
02_15	SMR	Concentration.	Concentration	No strategy used.	No strategy
02_17	SMR	Visual imagery of different pictures, such as a growing tree, its roots, people around the tree and so on.	Visual	Relaxation.	Relax
02_18	Gamma	Relaxation.	Relax	Maximal relaxation and breathing consciously. Remembering baseline period.	Relax
02_19	Gamma	Concentration on forehead.	Concentration	Concentration on forehead.	Concentration
02_20	Gamma	Visual imagination.	Visual	No strategy used.	No strategy
02_21	Gamma	Concentration and focusing thoughts.	Concentration	Concentration on moving bars, focusing and breathing.	Concentration
02_22	Gamma	Concentration on the bar in the middle of the screen and ignoring the left and right bar.	Concentration	Concentration on the bar in the middle of the screen and sitting calm.	Concentration
02_23	Gamma	Concentration on the bar in the middle of the screen and focusing on its size.	Concentration	Concentration on the bar in the middle of the screen.	Concentration
